# Identification of Novel/Rare *EWSR1* Fusion Partners in Undifferentiated Mesenchymal Neoplasms

**DOI:** 10.3390/ijms25031735

**Published:** 2024-02-01

**Authors:** Carmen Salguero-Aranda, Elena Di Blasi, Lourdes Galán, Laura Zaldumbide, Gema Civantos, David Marcilla, Enrique de Álava, Juan Díaz-Martín

**Affiliations:** 1 Instituto de Biomedicina de Sevilla, Department of Pathology, Hospital Universitario Virgen del Rocío, CSIC-Universidad de Sevilla, 41013 Seville, Spain; csalguero-ibis@us.es (C.S.-A.);; 2 Centro de Investigación Biomédica en Red de Cáncer, Instituto de Salud Carlos III (CB16/12/00361; CIBERONC-ISCIII), 28029 Madrid, Spain; 3 Department of Normal and Pathological Cytology and Histology, School of Medicine, University of Seville, 41004 Seville, Spain; 4 Istituto Nazionale dei Tumori, Università degli Studi di Milano, 20133 Milan, Italy; 5 Department of Pathology, Hospital Universitario Cruces, 48903 Barakaldo, Spain

**Keywords:** *EWSR1*, gene fusion, sarcoma, targeted RNA-seq

## Abstract

Recurrent gene fusions (GFs) in translocated sarcomas are recognized as major oncogenic drivers of the disease, as well as diagnostic markers whose identification is necessary for differential diagnosis. *EWSR1* is a ‘promiscuous’ gene that can fuse with many different partner genes, defining different entities among a broad range of mesenchymal neoplasms. Molecular testing of *EWSR1* translocation traditionally relies on FISH assays with break-apart probes, which are unable to identify the fusion partner. Therefore, other ancillary molecular diagnostic modalities are being increasingly adopted for accurate classification of these neoplasms. Herein, we report three cases with rare GFs involving *EWSR1* in undifferentiated mesenchymal neoplasms with uncertain differential diagnoses, using targeted RNA-seq and confirming with RT-PCR and Sanger sequencing. Two GFs involved hormone nuclear receptors as 3′ partners, *NR4A2* and *RORB*, which have not been previously reported. *NR4A2* may functionally replace *NR4A3*, the usual 3′ partner in extraskeletal myxoid chondrosarcoma. The third GF, *EWSR1::BEND2*, has previously been reported in a subtype of astroblastoma and other rare entities, including a single case of a soft-tissue tumor that we discuss in this work. In conclusion, our findings indicate that the catalogue of mesenchymal neoplasm-bearing *EWSR1* fusions continues to grow, underscoring the value of using molecular ancillary techniques with higher diagnostic abilities in the routine clinical setting.

## 1. Introduction

Sarcomas are a highly heterogeneous and complex group of rare cancers encompassing more than 100 different morphological entities that originate in the mesenchymal tissues, which include connective tissues, muscles, and bones. They represent about 1% of malignancies, with an annual incidence of approximately 50–60 new cases per million people/year, and account for 2% of mortality due to cancer [1]. Many of these entities are indeed exceedingly infrequent and have been defined as ultra-rare sarcomas (≤1 case per million people/year) [2]. Both this rarity and a remarkable histologic diversity pose major challenges for diagnosis, which can be further complicated by limited tumor tissue being available in biopsy specimens. Accurate identification of different sarcoma subtypes is crucial because of the serious consequences caused by inadequate treatment, as well as for patient prognostication since each entity requires specific treatment strategies and has different outcomes [3]. Precise diagnosis is also important for the accurate design of clinical trials testing new treatment regimens [4].

A large proportion of sarcomas are driven by oncogenic gene fusions (GFs) generated through the rearrangement of genetic material, usually via chromosomal translocations. They are, thus, designated as translocated sarcomas (t-sarcomas), typically occurring in adolescent and young adult populations and generally presenting as monotonous non-pleomorphic tumor cell proliferations. Most GFs are often associated with a particular histotype and are, thus, regarded as defining molecular genetic alterations, hence serving as optimal diagnostic biomarkers [5]. Many recurrent GFs in sarcoma involve transcription factors (TFs) or transcriptional regulators that become deregulated and fused to partner genes upon translocation to form chimeric TFs with new functional properties, leading to major changes in the transcriptional output of the cell [6]. Rearrangement of *Ewing Sarcoma breakpoint region 1* (*EWSR1*) was the first example of a cytogenetic abnormality in sarcoma. O. Delattre et al. reported the most frequent translocation t(11;22)(q24;q12) in Ewing sarcoma in 1992, producing the fusion between *EWSR1* and the ETS (E twenty-six) transcription factor *FLI1* (*Friend leukemia integration 1*) in their work [7]. The resulting chimeric protein fuses the N-terminal domain of *EWSR1* to the DNA-binding domain of *FLI1* or other related partners and functions as an aberrant TF with neomorphic properties that binds at GGAA microsatellite repeats, inducing active enhancers de novo and also repressing regulatory elements by means of different chromatin-remodeling mechanisms [8,9]. The N-terminal portion of EWSR1 provides the GF with a prion-like domain, an unstructured region with low complexity, which is prone to forming local aggregates, enabling the de novo formation of enhancers and the retargeting of chromatin regulatory complexes [10]. This favors the three-dimensional reconfiguration of chromatin interactions at looping hubs that control the activity of enhancers and promoters to induce oncogenic gene expression programs [11,12].

*EWSR1* stands out as the gene most commonly associated with translocations in sarcomas. This gene is implicated in a wide range of sarcoma subtypes, each characterized by distinct *EWSR1* gene fusions that engage with a diverse array of partner genes [13,14]. This promiscuity also occurs in Ewing sarcoma, in which *FLI1* can be replaced by *ERG* (5% of cases) or other ETS TF family members [15]. Undifferentiated small round-cell sarcomas (USRCSs) with *EWSR1* fused to non-ETS gene partners were formerly considered as neoplasms related to the Ewing family of tumors but are now recognized as distinct entities [16,17]. The most common representative of these non-ETS GF entities is *EWSR1::NFATC2* or *FUS::NTAC2* sarcoma, followed by *EWSR1::PATZ1* sarcoma. Though their morphological features resemble Ewing sarcoma, they present distinctive clinicopathological and molecular characteristics. For example, *EWSR1/FUS::NFATC2* sarcomas feature a marked male predilection, and *EWSR1::PATZ1* sarcomas can arise in a broad age range, in contrast to Ewing sarcoma [16,17]. Moreover, their transcriptomic and methylation profiles differ from Ewing sarcoma as well [18,19], reinforcing the concept of stand-alone entities. Fusions of *EWSR1* with the genes *SSX1*, *SSX2*, and *SSX3* have recently been identified in USRCSs. However, due to the limited number of cases, it is still uncertain whether these molecular hallmarks definitively characterize specific entities [20,21,22].

Other mesenchymal tumors characterized by specific *EWSR1* translocations include extraskeletal myxoid chondrosarcoma (*EWSR1::NR4A3*), desmoplastic small round-cell tumors (*EWSR1::WT1*), myxoid liposarcoma (*EWSR1::DDIT3*), and low-grade fibromyxoid sarcoma/sclerosing epithelioid fibrosarcoma (*EWSR1::CREB3L1/2*). Interestingly, the fusions *EWSR1::CREB* and *EWSR1::ATF1* arise in a number of mesenchymal tumors with different histologic features, such as clear cell sarcoma of the soft tissue, clear cell sarcoma-like tumors of the gastrointestinal tract (i.e., malignant gastrointestinal neuroectodermal tumors), angiomatoid fibrous histiocytoma, and primary pulmonary myxoid sarcoma. Moreover, beyond sarcoma, *EWSR1* GFs have also been described in a subset of mesothelioma and specific carcinomas and myoepithelial tumors [14].

Recently, a number of rare *EWSR1* partners were reported in a genomic study of tumor cases profiled with NGS panels, with most of them being sarcomas but with carcinomas also present [23]. Here, we describe three new examples of rare GFs in sarcoma, further expanding the growing catalogue of *EWSR1* partner genes that may define specific entities with distinct prognostic and therapeutic implications. These findings highlight the importance of molecular testing for the precise diagnosis and accurate clinical management of patients.

## 2. Results

### 2.1. Case 1 (EWSR1::NR4A2)

#### 2.1.1. Clinical and Pathological Features

A 32-year-old woman was referred to hospital with a 13 × 11 cm mass in the posterior compartment of her right thigh. She had noticed the mass for the past 2 years; it had been growing slowly and was without symptoms. Radical local excision was performed, with a diagnosis of low-grade extraskeletal myxoid chondrosarcoma (EMCS). Following the excision, the patient underwent brachytherapy, receiving a dose of 46.6 Gy (Table 1).

**Figure 1:**
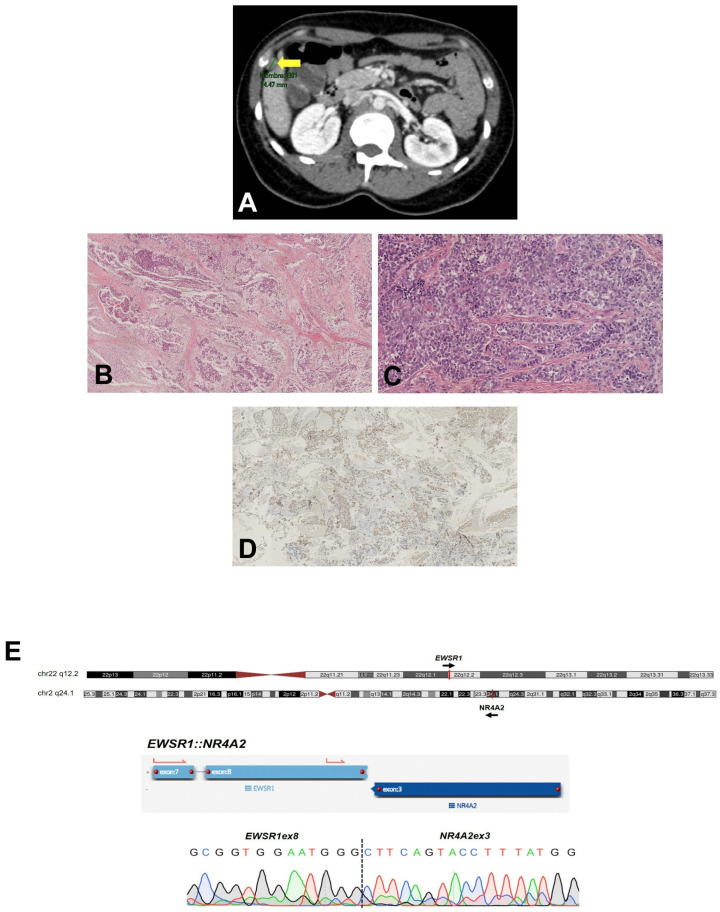
Case 1 (*EWSR1::NR4A2*). (**A**) MRI scan showing metastatic lesions in the peritoneum (indicated with a yellow arrowhead). (**B**,**C**) Representative images of the morphologic features of Case 1: The neoplasm was characterized by a lobular growth pattern with fibrous septa and myxoid stroma ((**B**), 4×); the cells were monomorphic, with eosinophilic cytoplasm and arranged in trabeculae and nests ((**C**), 20×). (**D**) Negative expression of S100. (**E**) Identification and confirmation of the gene fusion: Upper panel, graphical representation of chromosomes (ideogram) showing the cytoband location of the rearranged loci. Bottom panel, the gene fusion transcript is shown, as reported by the analysis software for targeted RNA-seq assays (see Section 4). Below, a DNA sequencing chromatogram shows the sequence of the breakpoint that was confirmed using RT-PCR and Sanger sequencing.

Nine months later, a metastasis was identified in the opposite thigh and surgically removed. Upon further analysis of the imaging, it became evident that this metastasis had, in fact, existed at the initial diagnosis but had been initially interpreted as a non-specific observation. Subsequently, three additional metastases were discovered a month later: one in the right-axillary soft tissue and two in the peritoneum (Figure 1A). The patient was enrolled in a clinical trial and received treatment with sunitinib and nivolumab. However, the treatment was discontinued after one year due to severe neurotoxicity. Despite this, the patient achieved clinical and radiological stability for 16 months until another metastasis in the chest wall emerged. The patient was lost to follow-up thereafter.

The initial diagnosis was conducted at a different hospital, and our pathology department conducted a thorough review before the patient’s enrollment in the clinical trial. At low magnification, the neoplasm exhibited a lobular structure embedded in an abundant myxoid stroma. The individual tumor lobules consisted of uniformly sized and shaped round cells, with ample eosinophilic cytoplasm and eccentric nuclei and arranged in cord-like patterns and trabeculae (Figure 1B,C). The main differential diagnosis, based on the morphological findings, included an EMCS and myoepithelioma.

#### 2.1.2. Molecular Findings

The FISH analysis, employing a break-apart probe, did not detect *NR4A3* rearrangements, ruling out the diagnosis of EMCS. Additionally, the lack of intense and diffuse S100 (Figure 1D) and cytokeratin immunoexpression did not align with a myoepithelioma diagnosis. The case was then tested with targeted RNA-seq, revealing a novel GF between *EWSR1* and *NR4A2*, an alternative partner that belongs to the same gene family of nuclear receptors as *NR4A3* (Figure 1E, Table A1). We confirmed the novel GF by means of RT-PCR and Sanger sequencing using gene-specific primers flanking the breakpoint sequence (Figure 1E). Subsequent FISH testing showed rearrangement of *EWSR1*. Consequently, the case was conclusively diagnosed as EMCS, featuring the novel *EWSR1::NR4A2* fusion.

### 2.2. Case 2 (EWSR1::RORB)

#### 2.2.1. Clinical and Pathological Features

**Figure 2:**
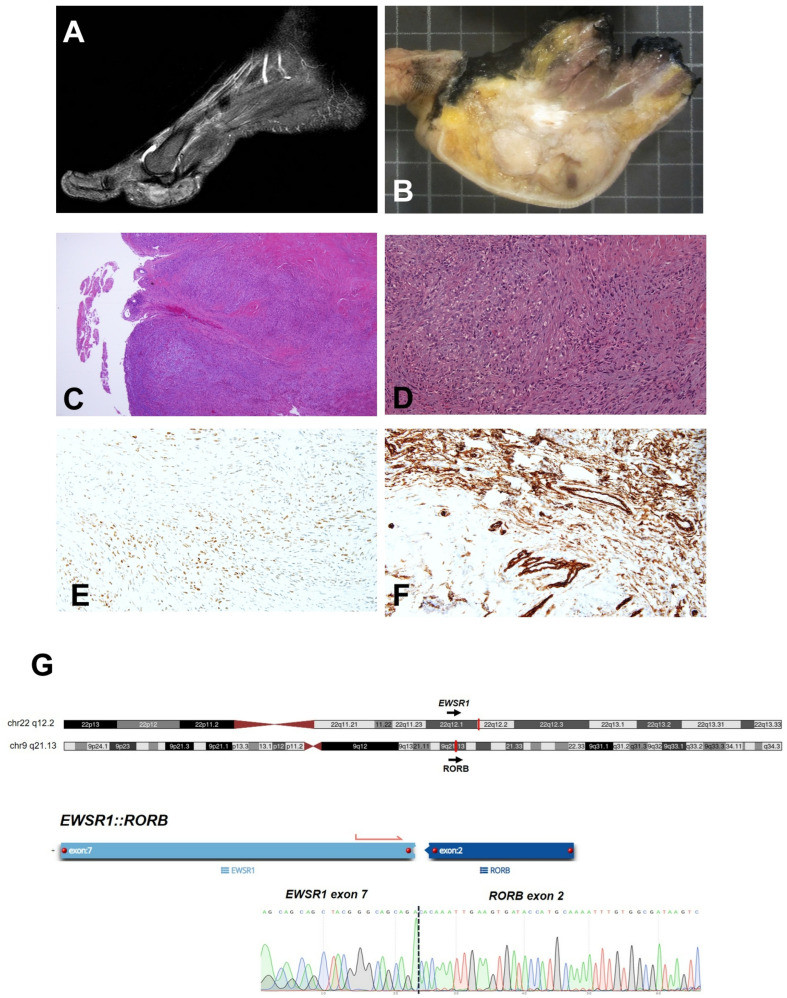
Case 2 (*EWSR1::RORB*). (**A**) MRI scan of the left foot of patient 2. (**B**) Representative gross image of metatarsal amputation. (**C**,**D**) Representative images of the morphological features of Case 2: The neoplasm was characterized by infiltrative pattern and solid growth, as shown at low magnification ((**C**), 4×), with heterogeneous neoplastic cells, both spindle and epithelioid ((**D**), 20×). The tumor specimen showed focal cytoplasmic positivity for TLE1 ((**E**), 20×) and CD34 ((**F**), 20×). (**G**) Identification and confirmation of the gene fusion: Upper panel, graphical representation of chromosomes (ideogram) showing the cytoband location of the rearranged loci. Bottom panel, the gene fusion transcript is shown, as reported by the analysis software for targeted RNA-seq assays (see Section 4). Below, a DNA sequencing chromatogram shows the sequence of the breakpoint that was confirmed using RT-PCR and Sanger sequencing.

A 61-year-old woman was evaluated for a nodule in the left foot plant at another hospital. Her medical history included arterial hypertension, type 2 diabetes mellitus, cholecystectomy, and an axillary lipoma. The patient reported that the slow enlarging nodule had been present for 2.5 years, accompanied by neuropathic pain in recent months. An excisional biopsy of the plantar nodule was performed, with an initial diagnosis of a giant-cell tumor of the soft tissue. After 6 months, a local recurrence prompted a referral to our institution. Imaging revealed a mass in the first metatarsophalangeal joint of the second toe, firmly attached to adjacent tendon structures (Figure 2A). The patient underwent metatarsal amputation of the first and second toes (Figure 2B) without any adjuvant therapy. Four years after amputation, the patient is still alive with no signs of local or distant disease at the last follow-up.

Both the primary excision and amputation were evaluated at our institution, the former as an external consultation. At low magnification, the tumor was characterized by an infiltrative pattern and solid growth. The neoplastic cellularity was heterogeneous and poorly differentiated, with spindle cells and epithelioid-to-ovoid cells with hyperchromatic or vesicular nuclei, with focal nuclear pleomorphism. Mitotic activity was low (up to nine mitoses in a 2 mm^2^ area) and only small foci of necrosis were found (Figure 2C,D). Immunohistochemical (IHC) staining only revealed focal cytoplasmic positivity for S100, TLE1 (Figure 2E), CD99, and CD34 (representative positive focal area in Figure 2F), which was regarded as non-informative, while melanocytic markers (SOX10, HMB45, and Melan-A), muscle differentiation markers (desmin and smooth muscle actin 1A4), vascular markers (ERG and CD31), mdm2, MUC4, STAT6, beta-catenin, and CD68 were negative; INI1 expression was retained.

#### 2.2.2. Molecular Findings

FISH analyses revealed *EWSR1* rearrangement, while SYT rearrangement was not detected, ruling out the diagnosis of synovial sarcoma. A subsequent targeted RNA-seq assay showed the novel translocation *EWSR1::RORB* (Figure 2G, Table A1), which had not been previously described, which was further confirmed using RT-PCR and Sanger sequencing (Figure 2G). The case was finally diagnosed as unclassified sarcoma of FNCLCC grade 2.

### 2.3. Case 3 (EWSR1::BEND2)

#### 2.3.1. Clinical and Pathological Features

**Figure 3:**
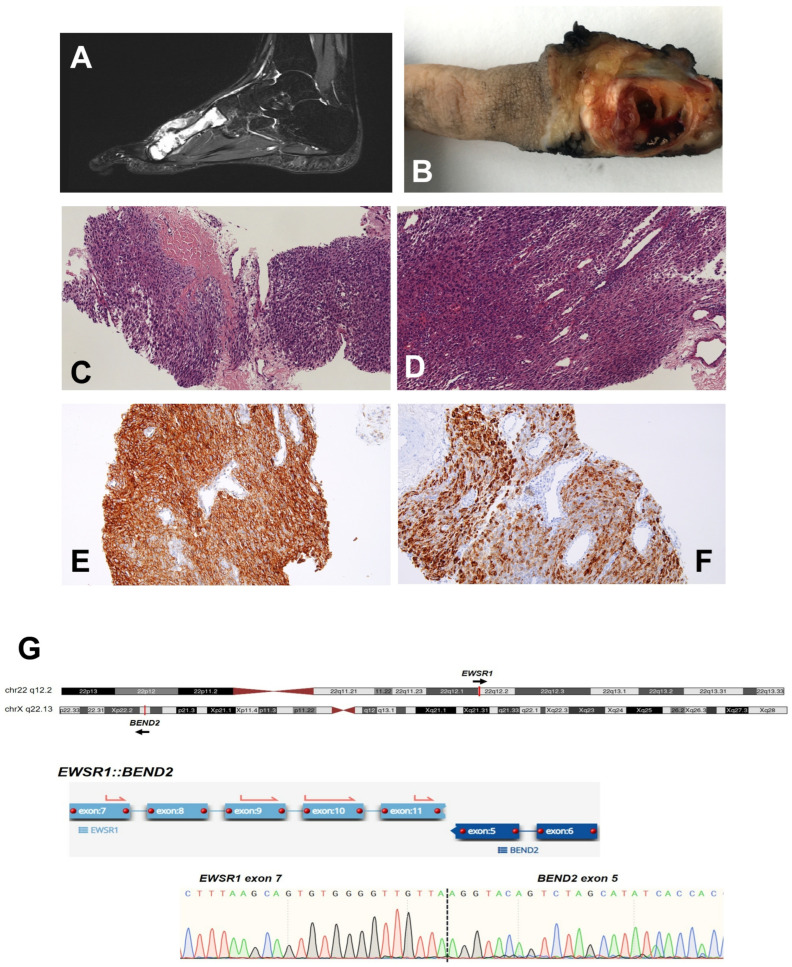
Case 3 (*EWSR1::BEND2*). (**A**) MRI scan of the left foot of patient 3. (**B**) Representative gross image of radical resection. (**C**,**D**) Representative images of the morphologic features of Case 3: The neoplasm was characterized by a solid, sheet-like growth pattern, infiltrating surrounding tissue ((**C**), 10×) without evidence of stroma and with dilated thin-walled vessels ((**D**), 20×). The tumor specimen showed diffuse and intense expressions of CD99 ((**E**), 20×) and EMA ((**F**), 20×). (**G**) Identification and confirmation of the gene fusion: Upper panel, graphical representation of chromosomes (ideogram) showing the cytoband location of the rearranged loci. Bottom panel, the gene fusion transcript is shown, as reported by the analysis software for targeted RNA-seq assays (see Section 4). Below, a DNA sequencing chromatogram shows the sequence of the breakpoint that was confirmed using RT-PCR and Sanger sequencing.

The patient was a 16-year-old female who presented with a pathological fracture of the second metatarsal of the left foot. She was assessed at a private center, where she was diagnosed with a bone cyst by means of radiography. Later on, she underwent an evaluation by a different traumatologist, who advised that an MRI scan be carried out. This MRI revealed a lytic lesion affecting the entire second metatarsal of her left foot, accompanied by a pathological fracture and a soft-tissue mass measuring up to 63 mm at its maximum dimension (Figure 3A). A biopsy of the lesion was performed with a diagnosis of USRCS at the external center. Neoadjuvant treatment was prescribed according to the SEHOP 2020 guidelines for the treatment of the Ewing sarcoma family of tumors. No histological response was observed (100% viable tumor), so the patient received adjuvant radiotherapy. Surgery was conducted to perform a radical resection of the second metatarsal bone in the left foot and a partial resection of the second cuneiform bone (Figure 3B). The patient is currently undergoing rehabilitation treatment, with good clinical condition and no signs of recurrence. Histological sections of both the presurgical biopsy and the tumor resection showed a neoplasm that infiltrated connective and muscular tissue, with a solid, sheet-like growth pattern, condensed around dilated vessels with thin walls, and without evidence of accompanying stroma or differentiation towards a defined mesenchymal lineage. The tumor cells were monotonous, small-to-medium-sized, and sometimes round, epithelioid, or even spindle-shaped depending on the area, with scant eosinophilic cytoplasm and ill-defined borders. The nuclei were ovoid, discretely elongated, and relatively isomorphic, with clefts and frequent nuclear pseudoinclusions, fine granular chromatin, and visible nucleoli. Twelve mitotic figures were counted per 10 high-power fields. No necrosis was observed (Figure 3C,D).

In the IHC study, tumor cells showed positivity for CD99 (Figure 3E), EMA (Figure 3F), vimentin, and TLE1, while they were negative for NKX2.2, WT1, BCOR, PDGFA, cytokeratins, muscle markers (smooth muscle actin, desmin, caldesmon, MyoD1, and myogenin), and melanocytic markers (HMB45, SOX10, and S100). Approximately 40% of the neoplastic cells were positive for D2-40. The immunophenotype of this neoplasm is, in fact, very unique. It is unusual for a round-cell sarcoma to have an intense, diffuse, membrane-patterned expression of CD99 and, at the same time, lack NKX2.2 expression. Non-Ewing round-cell sarcomas usually have a focal and patchy expression of CD99, on quite a few occasions showing simultaneous expression of NKX2.2 and/or WT1 or BCOR [16].

#### 2.3.2. Molecular Findings

FISH analyses showed an *EWSR1* rearrangement without alterations in *FLI1* and *SYT* (*SS18*) genes. The tumor was further evaluated using targeted RNA-seq in order to identify the gene fusion partner, revealing a fusion transcript *EWSR1::BEND2* between *EWSR1* exon 11 and *BEND2* exon 5 (Figure 3G, Table A1). The fusion event was predicted to be in-frame and was supported by 1977 reads spanning the breakpoint (Table A1). We confirmed this finding by means of RT-PCR, with specific primers flanking the breakpoint, and Sanger sequencing (Figure 3G).

## 3. Discussion

In this work, we have presented three cases with challenging or uncertain differential diagnoses that were ultimately resolved through the application of targeted RNA sequencing. This highlights the utility of molecular techniques with enhanced analytical sensitivity to single-gene tests in the diagnostic process of rare and heterogeneous entities, such as sarcomas. This is especially due to their multiplexing capability for the simultaneous screening of different events and their ability to identify novel GFs or novel fusion gene partners [24,25,26,27], as we have demonstrated here. Moreover, though conventional molecular testing and histopathology remain a diagnostic cornerstone, it is inarguable that new technologies such as NGS are contributing to improving the classification of a high variety of mesenchymal neoplasms. Recognition of potential novel entities through molecular characterization is essential to ensure their proper management. In this sense, NGS techniques help to define new diagnostic biomarkers based on molecular alterations or their consequences (i.e., GF target genes), which are much more specific than markers intended to determine the tissue of origin or the trend of differentiation [28].

**Figure A1:**
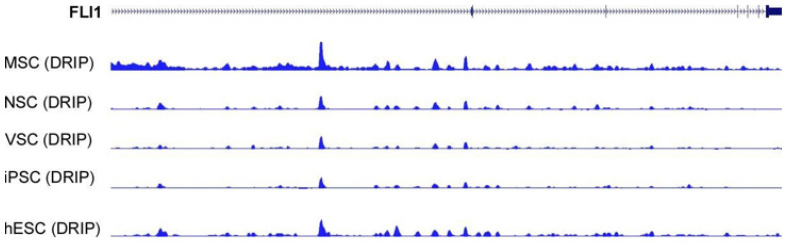
DRIP signal indicating that the presence of R-loops at the *FLI1* locus is increased in the mesenchymal stem cell lineage (MSC), which is the putative cell of origin of Ewing sarcoma. NSC, neural stem cells; VSC, vascular endothelial cells; ESC, embryonic stem cells; iPSC, human-induced pluripotent stem cells. Data were retrieved from Yan et al. 2020 [32].

The three cases we have presented expressed GF transcripts involving *EWSR1* as a 5′ partner and novel or rare 3′ partners, two of which had not been reported before. There seems to be an ever-expanding catalogue of genes that translocate with *EWSR1*, and interestingly, *EWSR1* GFs with uncommon partners are associated with a higher frequency of mutations in actionable genes [13,14,23]. Different *EWSR1* fusion partners endow the tumoral cells with specific properties, which, together with the cellular context and additional genetic alterations, determine the distinct biological features of each entity. Such promiscuity of *EWSR1* in translocation-driven sarcomas and other tumors may be explained by the high propensity to breakage of this locus in certain cellular contexts. It has been reported that highly active transcription of the *EWSR1* locus could induce the formation of R-loops that in turn favor double-strand breaks. Nicholas et al. [29], using prostate cancer cell lines, reported that androgen receptors promoted breakpoint generation in an intronic polyadenylation site of *EWSR1* near the Ewing sarcoma breakpoint hotspot, which was dependent on the formation of R-loops. Indeed, persistent R-loops are a direct cause of chromosomal rearrangements [30]. It is also tempting to speculate on the possibility that certain cellular contexts allow for specific loci rearrangements. In the case of Ewing sarcoma, the putative cell of origin of the tumor is a mesenchymal stem cell (MSC) in a specific stage of differentiation [31]. A recent study performing DRIP-seq (DNA–RNA hybrid immunoprecipitation) assays has shown that there is a differential enrichment of R-loops in different locations of the genome in stem cells of different lineages [32]. In this setting, R-loops cooperate with epigenetic marks in the differentiation process from pluripotent cells to different stem cell lineages [32]. Examination of these DRIP-seq data reveal that there is a differential enrichment in R-loops in the MSC lineage at the loci involved in the translocations described in Ewing sarcoma, such as *EWSR1*, *FLI1*, or *FUS* (personal observation, Figure A1 in Appendix A).

**Figure A2:**
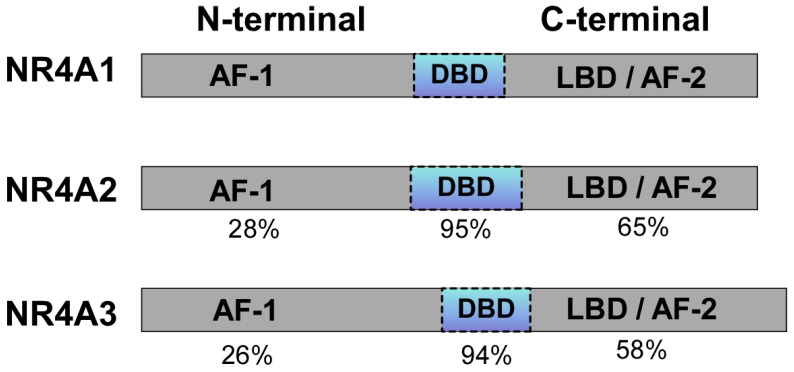
Schematic representation of the protein sequence of the three members of the subfamily of orphan nuclear receptors, NR4A. The percentages of amino acid sequence similarity in each domain compared with NR4A1 are indicated below the representations of NR4A2 and NR4A3. AF, activation function; DBD, DNA-binding domain; LBD, ligand-binding domain (adapted from Maruyama K. et al., 1998 [34]).

The case with ***EWSR1::NR4A2*** fusion presented histomorphological features highly suggestive of EMCS. However, single-gene testing by means of FISH with a break-apart probe for *NR4A3* did not support the diagnosis of EMCS. Targeted RNA-seq testing was key to resolving this discrepancy by revealing a new GF partner, *NR4A2* mapping in chr.2q24.1, which may provide equivalent functional properties to *NR4A3* to the chimeric protein. *NR4A2* belongs to the same family of orphan nuclear receptors as *NRA43*, the usual partner of the recurrent GFs defining EMCS [33]. We made sure that the GF call was not a consequence of a misalignment issue by manually inspecting the sequence, as NR4A2 and NRA43 are highly homologous. The three members of the NR4A family (NR4A1, NR4A2, and NR4A3) recognize common *cis*-elements, and thus, the chimeric protein could be deregulating similar target genes irrespective of the member of the family serving as the 3′ partner. They mostly diverge in the NH-terminal activation domains and the ligand-binding domain, while the DNA-binding domain (C4 zinc fingers) is highly conserved among the three proteins [34,35] (Figure A2). As occurs in the canonical GF of EMCS, the C-terminal RNA-binding domain of EWSR1 is replaced by the entire NR4A2 sequence in the predicted protein. NR4A receptors were initially described as immediate early-response genes induced by nerve growth factors [36]. Interestingly, transcriptional profiling of EMCS bearing different pathognomonic GFs (*EWSR1::NR4A3* and *TAF15::NR4A3*) revealed a different modulation of the axon-guidance and neurogenesis pathways through differential regulation of semaphorins and plexins [37]. Since these axon-guidance molecules establish crosstalk with MET and VEGFR, this might explain the observed activity of antiangiogenic drugs such as sunitinib and pazopanib in advanced EMCS cases [33]. In fact, the case we have presented here was enrolled in a phase I–II single-arm trial (NCT03277924) testing the combination of sunitinib plus nivolumab, but the treatment was discontinued due to the occurrence of toxicity.

The tumor with ***EWSR1::RORB*** fusion showed a morphologic appearance that was not distinctive of any specific type of round-cell sarcoma. The predicted chimeric protein contains the entire sequence of RORB (Retinoid-Related Orphan Receptor Beta), as the fusion involves exon 2 which is the first protein-coding exon. RORB is a member of the NR1 subfamily of nuclear hormone receptors. The RORB protein has two N-terminal C4-zinc finger domains that are able to bind DNA at hormone response elements, a C-terminal ligand-binding domain that can interact with transcriptional coactivators or corepressors, and two transcriptional activation domains, AF-1 and AF-2, with ligand-independent and -dependent activation functions, respectively. RORB expression was initially described to be restricted to the central nervous system, but it is also expressed in bone tissue, pancreatic and endometrial cancer, and uterine leiomyosarcoma. Its physiological functions include osteogenic repression during bone formation, retinal neurogenesis, and the regulation of the circadian rhythm. Interestingly, RORB-induced circadian rhythm abnormalities could be related to tumorigenesis, and RORB may also be involved in wnt deregulation in oncogenic processes [38]. To our knowledge, no GF involving *RORB* as a partner gene has been previously reported. Like NR4A genes, RORB belongs to the steroid/thyroid receptor gene superfamily, and hence, *EWSR1::RORB* may share common oncogenic properties with GFs involving these nuclear hormone receptors, or even with GFs involving *NCOA* genes that act as transcriptional coactivators for these nuclear receptors.

***EWSR1::BEND2*** fusions are recurrent in a specific subtype of astroblastoma that is closely related to ‘astroblastoma, *MN1*-altered′, an entity that harbors *MN1* GF with *BEND2* as the typical partner, but they are slightly different in their methylation profile [39,40,41,42,43,44]. Other reported cancers with *EWSR1::BEND2* include rare pancreatic neuroendocrine tumors, one case of a pancreas islet cell tumor, salivary carcinomas, and one case of an atypical carcinoid tumor of the lung [23,45,46,47] (Table S1). Regarding sarcoma, only one case with this fusion has been documented, a sinonasal spindle cell tumor, which exhibited histomorphological characteristics very similar to the case we have reported here [48] (Table S1). Additionally, a reported case of a soft-tissue tumor in the abdominal wall with the *MN1::BEND2* fusion presented a similar morphology as well [49]. Consequently, much like in astroblastomas, the *EWSR1::BEND2* and *MN1::BEND2* fusions may define a distinct subgroup of mesenchymal tumors. Intriguingly, our case showed intense and diffuse expression of CD99, a transmembrane cell adhesion protein routinely used for Ewing sarcoma differential diagnoses [15,50]. CD99 plays a role in antagonizing *EWSR1::FLI1*-induced neural differentiation through NF-κB signaling [51]. Thus, it would be interesting to investigate whether BEND2-rearranged mesenchymal tumors do express CD99 and its functional role. Interestingly, our case was refractory to the neoadjuvant treatment for Ewing sarcoma, suggesting the existence of major differences regarding tumor biology that would require different therapeutic regimes to be developed. Since *BEND2* and other BEN-domain-containing gene family members functionally interact with polycomb complexes to regulate transcription [52,53,54], targeting chromatin modifications could represent a new therapeutic approach worth exploring.

In summary, we have presented three cases with uncommon GF partners of *EWSR1* in sarcomas, with two of them being previously unreported. These findings add further evidence to the promiscuity of *EWSR1* in GFs in sarcomas and emphasize the value of technologies with high analytical sensitivity, such as targeted RNA-seq, to refine the classification of this heterogeneous group of entities. It remains to be confirmed whether these new alterations define specific entities, as occurs with similar findings in series with a very limited number of cases (i.e., *EWSR1::SSX* fusions [20]). Nevertheless, the detection of novel *EWSR1* GF not only provides new diagnostic markers but also opens new avenues for the identification of new therapeutic targets through full transcriptomic characterization of these novel entities. So far, USRCS with non-ETS fusions is usually treated with Ewing sarcoma regimes, and few studies have addressed their actual efficacy nor defined an optimal treatment strategy [55]. Since tumors with rare *EWSR1* GFs usually show a greater abundance of actionable targets than tumors with more prevalent *EWSR1* GFs [23], this could also be relevant for the clinical management of patients.

## 4. Materials and Methods

### 4.1. Clinicopathological Data

The three specimens were processed routinely and assessed as consultation cases at our hospital, which serves as a reference center for bone and soft-tissue tumors in Spain (Dept. of Pathology, Hospital Universitario Virgen del Rocío). The tumors were reviewed by experienced soft-tissue pathologists (E.D., L.G., L.Z., G.C., D.M. and E.A.), who evaluated the histopathological features, immunohistochemistry (IHC), and fluorescence in situ hybridization (FISH) (details of the antibodies, FISH probes, and protocols involved are available upon request). Clinical and follow-up data were obtained from the medical records in the electronic medical history with HUVR-IBiS Biobank (Hospital Virgen del Rocio—Institute of Biomedicine of Seville Biobank, Andalusian Public Health System Biobank). Table 1 summarizes all clinicopathological and molecular data for each patient included in this study.

This study was performed following the standard Spanish ethical regulations, and it was approved by the ethics committee of the Hospital Virgen del Rocío de Sevilla and the Fundación Pública Andaluza para la Gestión de la Investigación en Salud de Sevilla (FISEVI), Spain. Written informed consent was obtained from all patients, and all clinical analyses were conducted in accordance with the principles of the Declaration of Helsinki. 

### 4.2. Targeted RNA-Seq

NGS data from tumors were collected as assayed in the routine molecular testing using an Archer™ FusionPlex™ Sarcoma Panel. Total nucleic acid was extracted from FFPE tissue samples using an Agencourt Formapure Kit (A33341; Beckman Coulter, Indianapolis, IN, USA) following the manufacturer’s instructions. The isolated RNA was quantified using a Qubit™ RNA HS assay kit in combination with a Qubit^®^ 4 fluorimeter (Q32852; Thermo Fisher Scientific, Waltham, MA, USA). A total of 200 ng of RNA was used for targeted library preparation using the Archer™ FusionPlex™ Sarcoma Panel v1 or v2 (ArcherDX, Boulder, CO, USA) based on a targeted enrichment method called anchored multiplex PCR (AMP). Briefly, RNA was reverse transcribed using random primers, first-strand cDNA was synthesized, and RNA quality was assessed using the Archer PreSeq RNA QC assay. After second-strand cDNA synthesis, end repair, A-tailing, and adapter ligation, cDNA was amplified using two rounds of nested PCR using gene-specific primers. Final libraries were quantified with a KAPA Library Quantification Kit (KK4824; KAPA Biosystems, Wilmington, MA, USA) and pooled to equimolar concentration. Libraries were sequenced on an Illumina MiSeq with MiSeq 300v2 reagents (MS-102-2002; Illumina, San Diego, CA, USA) for paired-end reads, 150 base-pair reads, and dual-index reads. Samples were multiplexed such that each library was sequenced to at least 2 million paired reads or greater in depth. Demultiplexed FASTQ files were analyzed using Archer analysis pipeline v7.1.0-14. A minimum of five reads with three or more unique start sites spanning the breakpoints were set as cutoffs to call fusions. Sequencing data and parameters are shown in Table A1 in Appendix A.

### 4.3. RT-PCR and Sanger Sequencing

Primers flanking the breakpoint sequence were designed with Primer3Plus [56] according to the reads from RNA sequencing. First, 450 ng of RNA (same preparation procedure as that used for targeted RNA-seq) was retro-transcribed using a High-Capacity cDNA Reverse Transcription Kit (Thermo Fisher Scientific). PCR was performed with Q5^®^ Hot Start High-Fidelity 2X Master Mix (New England Biolabs, Ipswich, MA, USA) and specific primers. The PCR products were analyzed using gel electrophoresis and purified using a QIAquick PCR Purification Kit (Qiagen, Hilden, Germany). Direct Sanger sequencing of PCR products was performed using BigDye Terminator v3.1 chemistry.

Primers for the amplification of fusion transcripts were as follows: *EWSR1::NR4A2*; primer forward: AGGGGAAGAGGGGGATTTGA, primer reverse: GGACAGGGGCATTTGGTACA. *EWSR1::RORB*; primer forward: CCTACAGCCAAGCTCCAAGT, primer reverse: CCAGAGGACTTATCGCCACA. *EWSR1::BEND2*; primer forward: TCTTGATCTAGGCCCACCTG, primer reverse: TTCATGACATGCTGCTGACTC.

## Figures and Tables

**Table 1:** Clinicopathological features.

Case	Sex	Age at Dx	Primary Tumor Location	Histologic Pattern	Original Diagnosis	Treatment	Outcome
#1	F	32	Posterior right thigh	Round cells in myxoid stroma	Extraskeletal myxoid chondrosarcoma	Primary tumor: resection + RT Metastatic recurrence: sunitinib + nivolumab	AWD
#2	F	61	Foot sole	Solid spindle cells and epithelioid-to-ovoid cells	Giant-cell tumor	Primary tumor: resection Local recurrence: metatarsal amputation	ANED
#3	F	16	Second metatarsal of the left foot	Solid round, epithelioid, or spindle cells	Bone cyst	Primary tumor: neoadjuvant CT with no effect + radical resection	ANED

ANED, alive with no evidence of disease; AWD, alive with disease; Dx, diagnosis; F, female; M, male; CT, chemotherapy; RT, radiotherapy.

## Data Availability

The data presented in this study are available from the corresponding author upon request.

## Supplementary Materials

The following supporting information can be downloaded at https://www.mdpi.com/article/10.3390/ijms25031735/s1.

## Appendix A

**Table A1:** Sequencing data and parameters.

Case	RNA-Seq Panel	Fusion Transcript	Transcript Accession	Breakpoint (GRCh37–hg19)	Total Reads	Fusion Reads (% Primer Reads)	Unique Start Sites	QC
#1	FusionPlex Sarcoma v2	*EWSR1ex11::BEND2ex5*	*EWSR1* NM_001163285.1 *BEND2* NM_153346.4	chr22:29688595, chrX:18222035	2,091,797	1977 (35.9)	207	116.7
#2	FusionPlex Sarcoma v1	*EWSR1ex7::RORBex2*	*EWSR1* NM_001163285.1 *RORB* NM_006914.3	chr22:29683123, chr9:77245198	2,014,755	156 (23.3)	48	78.7
#3	FusionPlex Sarcoma v1	*EWSR1ex8::NR4A2ex3*	*EWSR1* NM_001163285.1 *NR4A2* NM_006186.3	chr22:29684775, chr2:157186522	2,321,509	142 (14.3)	58	99.7
